# Hospital and Institutional News

**Published:** 1915-03-13

**Authors:** 


### HOSPITAL AND INSTITUTIONAL NEWS.

### A VISIT IN STATE TO COVENTRY AND WARWICKSHIRE HOSPITAL.

The High Sheriff of Warwickshire, Colonel W. F. Wyley, accompanied by his chaplain, the Rev. Canon Baillie, and Mrs. Wyley, paid an official visit in state to the Coventry and Warwickshire Hospital last Saturday. Colonel Wyley drove from his residence, the Charterhouse, in the official coach, which had been specially brought over from Warwick, and was preceded by two mounted constables. His arrival at the hospital was announced by two heralds, and the party was received by the chairman of the hospital, Mr. W. J. Wormell; Mr. Harold Twist, vice-chairman; Mr. Edward M. Iliffe, Dr. Hawley, Dr. Webb Fowler, Mr. H. H. Ilett, chairman of the Hospital Saturday Committee; Mr. E. Marriott, Dr. Masterman, and Dr. Rice, house surgeons; Miss Hutchinson, matron; and the secretary, Mr. J. A. Rudd. The party visited all the wards of the institution, and in the soldiers' wards, which have been specially provided for the accommodation of sick and wounded, Colonel Wyley spoke to each patient. In the Alfred Herbert ward he wished the patients, in the name of Warwickshire, a speedy recovery to health. The High Sheriff on the occasion of his visit made a special donation of twenty guineas to the funds of the institution. Before leaving, the chairman cordially thanked Colonel Wyley on behalf of the committee of the hospital for so kindly paying an official visit to the institution before relinquishing his office as High Sheriff of the county.

### THE GERMAN ATTACK ON A HOSPITAL SHIP.

A remarkable admission of the attack on the hospital ship *Asturias*, off Havre, is published by the German Embassy, according to a Reuter statement in the *Times*. The details of the admission are as follows: "The Government is sorry to admit that the *Asturias* was attacked on February 1, at 5 p.m. Looming up in the twilight, carrying the lights prescribed for ordinary steamers, the *Asturias* was taken for a transport carrying troops. The distinctive marks showing the character of the ship not being illuminated, they were only recognised after a shot had been fired. Fortunately the torpedo failed to explode, and the moment the ship was recognised as a hospital ship every attempt at further attack was immediately given up." It is satisfactory to learn that a mistake has been made, and also frankly admitted, and it is also welcome news to learn that whatever other usages of international law may be overridden in active warfare, the inviolability of the hospital ship is still upheld by all combatants.

### BLACK-AND-WHITE SIGNS FOR HOSPITALS.

The subject of hospital flags in time both of peace and war has again lately been alluded to in our columns, and it is therefore interesting to note a communication bearing on the subject which has been sent by the Home Secretary to the Town Clerk of Leeds. The communication states that, in the event of a bombardment or the landing of hostile troops, civil hospitals (a term used to cover mental hospitals, buildings devoted to charity, churches and museums, and historic monuments) should be marked by a stiff rectangular panel, divided diagonally into two pointed triangular portions, the upper part being black and the lower white. The Red Cross flag, it is pointed out, may be used only by hospitals which are exclusively under naval or military control, or in cases where special authority has been granted by the Army Council. It would be interesting to know to what extent, if any, the buildings displaying it would be protected from bombardment or attack. In view of the facts already known about bombardments during the early months of the war, we fear that there is not much chance that any sign will serve much protective purpose, or that care would be taken to avoid shelling the building on which it is used. The aspect of the matter in international law is as follows: Under Article 27 of the annex to the Hague Convention of 1907 it is provided that such buildings as those enumerated above are to be spared as much as possible during bombardments, provided that they are not at the time being used for military purposes. Such buildings, it is also stated, should be clearly indicated by distinctive and visible signs which have been notified beforehand to the enemy. The panel above mentioned is then described, and it is pointed out that the Red Cross may be used only by hospitals exclusively under military or naval control. That is the theory of the matter. We hope that English institutions will have no experience of the practical value or otherwise of the black-and-white panel.

### THE NEW CHILDREN'S HOSPITAL, BIRMINGHAM.

Probably the institution whose most ambitious scheme has been interfered with by the war is the Birmingham and Midland Free Hospital for Sick Children. The committee had calculated that the new building would be ready by June 30, when the hospital's president, Mr. Edward Cheshire, had some reason to hope that the King would pay a visit and perform the opening ceremony. But as it is, not only is this hope disappointed, but the difficulty in providing men and material is delaying the actual work, while the clearing off the balance on the building fund is now more difficult than ever, and the £13,000 to be found for this purpose does not include the sum which is asked for in connection with a new out-patient department on the same site, which is held to be essential to the hospital's efficiency by the committee and the medical staff. It has been found impossible to proceed with this part of the scheme. This fact is proving a double disappointment, since the medical committee report that "an enormous number" of out-patients requiring admission are unable to be taken in. Birmingham is such a centre of institutional enterprise, however, that we hope it may yet prove what is still the fact, that the resources of charity are very far from being exhausted.

### CHAIRMAN OF THE WEST KENT HOSPITAL RESIGNS.

An epoch in the development of the West Kent General Hospital closes with the resignation of Mr. G. Marsham from the chairmanship, a post which he has filled for twenty-nine years. It is characteristic of the chairman's point of view that he should attribute the success which has attended the hospital during his period of office to the balance which has been held between the committee, the medical staff, and the nursing department. This always means in practice that there is easy, if informal, means of communication between these three branches of hospital work. During Mr. Marsham's chairmanship many developments have occurred. The Hallowes Ward, endowed by Lady Howard de Walden, was added in 1890, and three years later the isolation block was built. Then came the Diamond Jubilee of 1897, when it was decided, here as in so many other institutions, to commemorate the occasion by an addition to the hospital. At the West Kent General Hospital this extension included a chapel and a new out-patient department. Not long passed before a new operating theatre was added, and afterwards came the further need for a new special department, which with an $x$-ray installation has been recently added, thanks to a generous gift from Sir Marcus Samuel. A memorial to King Edward took the form of a special fund, and a system of district nursing was established. The successful expansion of usefulness here summarised has, however, depended not only on a committee of loyal workers and a generous public, but also on the lead and example of the resigning chairman, who has been generous in gifts of money in addition to personal service in the hospital's cause.

### A GRADUATED PAY HOSPITAL.

The feature of the Highfield Hospital is the system of graduated charges to suit the means of its patients, some facts of which were disclosed at the recent annual meeting presided over by the Mayor of Leicester, Alderman North. The report presented showed that the number of patients had increased from 109 to 124. The income from patients' payments had increased to £598. Owing to the generosity of Lord Dysart, a gift of £70 and a challenge was the means of discharging the adverse balance of 1913 at the bank, although it was regretted that there was at the close of the year 1914 a deficit of £229, due largely to causes incidental to the war. Reference was made to Sir Henry Burdett's visit earlier in the year, and to his observations and commendation of the "thrift lines" on which the hospital was administered. Acknowledgment was made of the generous gift of a shelter for the grounds, at a cost of £64, by Dr. Clifton, one of the medical staff, in celebration of his seventy-sixth birthday.

### KITCHENER'S INDIAN HOSPITAL.

Kitchener's Hospital for wounded Indian soldiers at Brighton has now been opened. This is in addition to the Indian hospitals already established in the town at the Dome, Pavilion, Corn Exchange, and York Place School. It contains 1,700 beds, with the possibility of increasing to another thousand. The wards vary in size from small ones containing eight beds to large ones with fifty beds. There are an $x$-ray department, operating theatres, a pathological department for the examination of specimens and for the preparation of vaccines, etc., an electro-therapeutic department, disinfecting rooms for clothes, wards for infectious cases, and special rooms for mental cases. The nursing is done by men of the R.A.M.C. or by Indian native orderlies. The staff consists of picked men of the Indian Medical Service under the command of Colonel Seton, of Simla, to whose organising ability the equipment of this hospital is due.

### MINERAL-WATER TREATMENT FOR OFFICERS.

We have too often upheld the claims of British spas to the attention of the public not to note with satisfaction the use which is being made of the Bath waters, which were placed at the disposal of the War Office and the Admiralty some time ago. The offer was accepted, and wounded officers from the Front have been taking advantage of the mineral-water treatment, with the result that many have been able to return to active service. The accommodation at Bath is being increased by the opening of a convalescent home for officers. This activity should serve the double purpose of giving many officers a water treatment likely to be valuable to them, and of increasing the numbers of those informed by experience of the value of British spas. We may be sure that those who have benefited will not forget the fact, and the numbers now attending Bath should serve to spread its reputation and help to build up an intelligent public opinion on the value of our home spas, waters, and bathing establishments.

### THE LATE DR. S. H. HABERSHON.

We regret to record the death, at the early age of fifty-seven, of Dr. Samuel Herbert Habershon, M.D., F.R.C.P., the senior physician to the Brompton Hospital for Diseases of the Chest. A specialist on these diseases, who contributed various interesting studies on different aspects of his subject, including a remarkable work on "Diseases of the Stomach," Dr. Habershon was much respected in the profession, and his advice as consultant was naturally much sought by a wide circle of patients. Educated at St. Bartholomew's Hospital, he graduated both in arts and medicine at Cambridge, taking his M.D. in 1887 and becoming an F.R.C.P. in 1891. Apart from his medical work, which was scholarly and valuable, Dr. Habershon possessed a singular charm of manner and gentleness of character which endeared him to his friends and patients. His recreations were the recreations of the scholar and cultivated amateur, and he was a keen musician. Every aspect of his subject interested him and engaged his attention, including climatology, of which he made quite a study. He will be missed by many as a friend and a scientist, whose life embodied without ostentation the best traditions of medical learning and practice.

### A TESTATOR'S PROVISION FOR A NURSE.

BEQUESTS to medical men and nurses from grateful patients are not of such frequent occurrence as not to merit a passing record, and a notable instance is provided by the will of the late Mr. Lewis William Novell, who died at the age of sixty-eight at a London hotel on December 23, 1914, leaving an estate of the gross value of £42,078. Among other bequests was the sum of £3,500, and an annuity of £25, together with certain personal effects to his nurse, Miss Ellen Freeborn, who thus benefits by what amounts to an annuity of some £150. Except in those cases in which a grateful patient bequeaths a substantial part of his fortune to his medical attendant or nurse, the sum left does not often amount to the above figure. Indeed, if we make a practice of glancing down the list of wills and bequests published in the newspapers we may be sometimes surprised at the comparatively small sums which testators bequeath to faithful servants, retainers, and others who have spent many years in their service. It is, therefore, pleasing to record the above instance, which shows that skilled service and medical attention are appreciated, and is doubly welcome in that one of the difficulties of the nursing profession is to find the means to make adequate provision for old age when the time comes to retire from active practice. Another bequest to a nurse is recorded in the Note headed "£25,000 for a Cottage Hospital."

### CENTRAL STORES FOR POOR-LAW AUTHORITIES.

THE Southwark Board of Guardians have decided to approach the Local Government Board asking whether it would not be possible to start a central stores under the control of the authorities at Whitehall or of some other public body, from which the various Metropolitan Boards of Guardians could draw the requisite supplies for their various institutions. There have been grounds for complaints against numerous contractors during the past few months. It was stated at one of the Southwark institutions that the coal had run down to the last ton, and the Guardians had to buy house coal at 33s. a ton in order that the old people should be kept warm. They had not many days before given their contractor a substantial increase, and he was asking for a further one, which later they were forced to give. They did not refuse lest the infirmary might have been left without coal. Other goods have had to be obtained in a similar manner. Whether the establishment of central stores will remove all the evils is doubtful, but it might result in more equal distribution amongst the various institutions in the Metropolis. Whilst one Board is unable to get coal, another is storing 400 tons simply because the Guardians have acceded to the extreme requests of their contractors. It is thought that such unequal distribution would be stopped by all the Poor-Law authorities obtaining their supplies from a central store. What are our readers' opinions?

### BAYONETING AN ARMY SURGEON.

IT is well to regard with caution the stories always current in time of war concerning the atrocities perpetrated by the enemy with which the nation is fighting, but when allegations are made concerning the abuse of the Red Cross, or the bayoneting of medical officers, they may be recorded without prejudice when they come from what apparently is a first-hand source. In this connection a letter from an officer of the Royal Army Medical Corps has been published, which professes to give an account of a grave violation of the Red Cross. The writer, who is stated to be in charge of an Indian hospital, is quoted as follows:—"One case was an assistant surgeon with one bullet and three bayonet wounds. He was left behind with some badly wounded men in a trench. When the Germans came up he pointed to his Red Cross. The officer in charge went away to consult with another, and then returned and shot him at five paces. The bullet ran round his ribs. Seeing that he was not dead, the Germans then bayoneted him three times and left him. He is now on his way back to India and won't be much the worse." This story bears on the face of it no excuse for the facts which it alleges, and our readers will recall the statement made some weeks ago in our own columns by another officer of the Royal Army Medical Corps, who declared his belief that there was no doubt that the Germans had used the Red Cross as a means to cover attacks "on our fellows," and added a strong denunciation of their methods.

### CHILD WELFARE WORK AT BRADFORD.

THE development of that new subdivision of public health known as child welfare, mothercraft, and day nurseries is well seen in the first medical appointments to be made under a scheme of this class at Bradford. The object of the scheme is to fill up gaps existing in the present medical supervision, which includes infant consultations, dealing with children up to a year old, and the school clinic for those between the ages of five and fourteen, after which the National Health Insurance Act is supposed to cover all existing medical needs, so far as the public conscience is awake concerning them. Under the new scheme the children who are too old to benefit by the infant consultations and too young for the school clinic—that is to say, who are between the ages of one and five years old—will have medical care within their reach. To carry this out a special sub-committee of the Bradford Health Committee have appointed two medical officers and seven lady health inspectors. The medical officers, Dr. Sophie B. Jackson and Dr. Grahame H. Skinner, receive a salary of £350 a year each. The former, in addition to holding public health and school appointments at Croydon and Leeds, has had experience in the children's hospitals of Berlin and Vienna. As far as the children of Bradford are concerned, the scheme of medical supervision up to the age of sixteen appears to be complete, and the experience gained will be of value to other cities not so elaborately organised in this important respect.

### BIRMINGHAM POOR-LAW INSTITUTIONS FOR THE WOUNDED.

Evidence of future military operations is to be found in the preparations being made for further accommodation among Poor-Law and other institutions not previously made use of for this purpose. Last week at a conference of representatives of Poor-Law Unions in the neighbourhood of Birmingham it was announced that the War Office had asked the Local Government Board to arrange for one or more of the Birmingham Poor-Law institutions to accommodate wounded soldiers. It was explained by Mr. Curtis, Birmingham Union, that either the largest and best-equipped institution should be entirely used for this purpose, or two adjoining institutions. Such a proposal, he added, was impracticable unless the neighbouring Unions, by pooling their spare accommodation, could receive boarders from Birmingham Union, and unless the latter could also be relieved of its phthisical cases now in the infirmaries. He understood that the cost of the cases boarded with other Unions would be repaid by the Government. After discussion, it was unanimously decided by the representatives to recommend their respective Boards to receive boarders from the Birmingham Union to the limit of spare accommodation, so that the War Office might have exclusive use of one or more of the city's institutions. It is perhaps not too much to see in these proposals the beginning of a closer scheme of co-ordination between the different Poor-Law institutions, and although Birmingham has always been a pioneer in this respect, events are moving generally in this direction, and it will not be easy to put the clock back at the conclusion of the war.

### EXTENSION OF CROYDON MENTAL HOSPITAL.

Only last week we drew attention to the alleged overcrowding in the Lancashire mental hospitals, and the reverse side of that question—namely, the need for extension—is a matter of urgency at the Croydon Mental Hospital, Warlingham. So much overcrowded is the male side of the institution that, according to the medical superintendent's report, it is practically impossible to take in any more patients. At the end of last month there were, excluding boarded-out patients, twenty-eight in excess of the normal number. "In some of the wards," reported the medical superintendent, "the beds are packed so closely that it is impossible to move between them." On an average each of the large wards has seven or eight beds apiece more than its normal number. It is pointed out in the same report that additional day room is required in any extension over and above normal additions in this respect. Additional accommodation sufficien to satisfy probable needs for ten to fifteen years is suggested, and the available space is deemed sufficient to accommodate some 200 more patients. After such an extension the patients would number about 900, for whom at least one other assistant medical officer would be required. When the institution was built the accommodation for the hospital's staff was planned on the basis of there being 450 patients, though kitchen and stores were designed with a view to extension. It is, therefore, necessary to supplement the accommodation and quarters of the matron, the assistant matrons, the married attendants, and so on. The visiting committee adopted the above report and sought the authority of the Council to have plans and estimates prepared for an extension sufficient to provide for 200 more male patients and to engage the services of an architect to supervise the plans. At the same meeting at which this report was passed it was decided to raise the salary of the chaplain, the Rev. H. M. Scott, from £65 to £100 per annum, and that of the second assistant medical officer from £250 to £275 per annum, with emoluments.

### EX-PATIENTS AS HOSPITAL SUBSCRIBERS.

A few years ago it was decided to make an attempt to raise funds for the benefit of Batley Hospital by means of a gathering of ex-patients—that is to say, to secure gifts from those who had had personal experience of the benefits of the institution. This attempt has now crystallised into an annual function, the success of which may be judged from the fact that a cheque for £100 from the ex-patients' dance was lately handed over to the hospital's treasurer, Mr. F. W. Akeroyd. The secretary of the ex-patients' committee, Mr. J. Webster, at a recent meeting recalled the first occasion on which the ex-patients' committee had met, and said that none could work more successfully for the hospital than those who knew its value as actual patients. Every one present at the gathering had been in hospital, and the main means which has been adopted for raising money has been an annual subscription dance organised by the ex-patients. The number of beds in Batley Hospital is forty-five, and it is interesting to see how successful has been this, we will not say novel, but unusual means for raising money. No doubt the possibility of attracting voluntary support from ex-patients is somewhat dependent upon the nature of the district which any particular institution serves, but what is possible by this means is well shown in the above case.

### ROYAL ALBERT EDWARD INFIRMARY, WIGAN.

A comprehensive new out-patient department, which will include accommodation for various special treatments, will shortly be added to the Wigan Infirmary. In 1911 plans were first prepared for this purpose, but delays occurred, and in the meantime, as a result of a series of visits paid to other institutions, the plans were modified so as to secure greater economy by centralisation and co-ordinating the new buildings with the old. In this way the difficulty created by the rise in price of building materials was partly met. Thus, with the beginning of March the tender for the erection of the new premises sent in by Messrs. Maisey Brothers, of Pemberton, was accepted. The cost is £12,000, and the firm responsible for the plans is that of Messrs. W. C. Ralph and Son, of Wigan. The new department will comprise a central waiting room, with consulting, $x$-ray, bath, and sterilising rooms, and a dispensary. A refreshment bar is also to be provided, where coffee and light refreshments are to be sold to waiting patients. The ground floor consists of a central hall with consulting rooms grouped about it, a small theatre, and a receiving room, which is provided with a separate approach from the grounds, and is connected by a covered corridor to the main block, so as to provide easy access for the removal of accident cases to the main building. An appeal which had been drafted was withdrawn on the outbreak of war, and as debt of about £15,500 was recorded in the last annual report, it is a matter of grave importance that the new extension should meet with not merely hearty, but very generous support. As, however, the hospital placed fifty beds at the disposal of the authorities for the benefit of wounded soldiers, there is a unique opportunity, if it is properly made use of, for raising funds, since the war, far from exhausting the stream of charity, has been chiefly remarkable as showing apparently inexhaustible and unsuspected sources of charity.

### EMERGENCY ANNUAL SUBSCRIPTIONS.

An idea which may possibly prove suggestive is to be found in the conditions attached to a gift recently received by the trustees of King Edward's Hospital Fund for London. A cheque for £500 has been received from Sir John Ellerman, Bart., which has been given as an annual subscription so long as the war lasts, and which will be withdrawn only "a year or two after the war, when things become normal again." The idea thus is to assist the hospitals over a difficult period. There is nothing like making a strong point out of the weakest joint in your armour, and emergency annual subscriptions, as they may be called, might well commend themselves at all events to wealthy supporters of hospitals, if the suggestion were brought personally to their notice, and were enforced by Sir John Ellerman's example. At all events his motives are worth emphasising, and we cannot but believe that they will commend themselves to others, in which hope the details of his gift are recorded here.

### AIDING THE HOSPITALS' WORK FOR THE WOUNDED.

A very successful Sale of Work in connection with the Voluntary Hospitals Brigade was held at Camberwell House on Tuesday, March 2, Dr. Edwards, the medical superintendent, having kindly granted the use of three of the large day-rooms for the purpose. The opening ceremony was performed by Mrs. Dhunjibhoy Bomanji, of Bombay and Oakley Court, Windsor. In the course of her speech Mrs. Bomanji called attention to the objects of the Brigade, and asked those present to assist the hospitals not only by their contributions at the moment, but also by undertaking to raise at least £1 a year during the time the war lasted. "Many special funds had been started and generously subscribed to," she said, "but there had been a tendency to overlook the pressing needs of the voluntary hospitals at a time when their necessity was greater than ever." In addition to the sale of goods, most of which were made by members of the staff and by lady patients, there were concerts and an organ recital in the new chapel; while the refreshment room was well patronised, and yielded quite a substantial profit. The various artists kindly gave their services for the occasion. The organisation of the proceedings was carried out by Mrs. H. J. Norman, wife of the senior assistant medical officer, who was assisted by the matron and the staff. It will be remembered that the proposal to start a Voluntary Hospitals Brigade was formulated in The Hospital in its issue of November 28 last. The response was remarkable and cheering, and, as announced in these columns on February 20, with a view to avoiding the undesirable multiplication of organisations for raising funds in connection with the war, the work thus initiated is to be carried on through the League of Mercy, which is collecting funds to assist in defraying the cost of treatment of the sick and wounded in our voluntary hospitals.

### £25,000 FOR A COTTAGE HOSPITAL.

A very remarkable residuary estate, expected to amount to £100,000, has been left by Mr. Edward Wright, solicitor, of Leamington, in equal shares to two hospitals and two other charities. The following is a summary of his will: Mr. Edward Wright, who was lately chairman of the Leamington Priors Gas Company, and died on January 16 last, aged eighty-five years, left estate of the gross value of £141,249. The testator left £1,000 each to the Royal Hospital for Incurables, Leamington, the Warneford and South Warwickshire Hospital, and to the Ellen Badger Cottage Hospital, Shipston-on-Stour. He also left £100 each to his two nurses. After setting aside stocks of the face value of £4,000 upon trust for his brother for life, and making several other bequests, the testator left the residue of his property, which, it appears, will amount to over £100,000, equally between the Warneford and South Warwickshire Hospital, the Ellen Badger Cottage Hospital, Shipston-on-Stour, and two other charities. It would, therefore, seem that the ultimate share of each of these institutions will be £25,000, a sum which probably forms a record so far as a cottage hospital is concerned. It is also noteworthy that this will, which shows such regard for voluntary hospitals, contains, as above mentioned, two small legacies for nurses.

### CONTRIBUTIONS FOR SOLDIER PATIENTS.

It is well, as a matter of history, to place on record the questions and answers which passed in the House of Commons last week on this subject, although the point as to whether a difference would be made in the rate paid to the general hospitals in the Metropolitan area and the hospitals throughout the country was not made clear. Mr. Lawson asked the Financial Secretary to the War Office what was the basis upon which contributions were made to the voluntary hospitals which, with the approval of the War Office, were admitting wounded soldiers; and whether such basis was uniform throughout the United Kingdom, or whether a varying scale had been adopted; and, if so, upon what principle the variations had been made. Mr. Baker, in reply, pointed out that the use of the voluntary hospitals had not been offered on a commercial basis, and that the assistance given from public funds had taken the form of a grant in aid on the basis of a daily rate per occupied bed. This rate, he added, was normally 2s., but in some cases a somewhat larger grant might be given by the general officer commanding. On being asked whether a difference would be made between the general hospitals of the London district and the provincial hospitals, Mr. Baker replied that he could not answer the question. Perhaps the chief satisfaction to be obtained from these replies is the official admission of the uncommercial basis on which the voluntary hospitals are doing the country's work, for this should serve to consolidate the generosity of all hospital supporters.

### "THE EDUCATION OF THE HOSPITAL OFFICER."

We publish in another part of this issue an interesting article on the above subject, written by Mr. George Watts, the secretary of the City of London Hospital for Diseases of the Chest. In this, while discussing the best means for improving the training, in the widest sense, of junior hospital officers, he outlines certain proposals for a system of examinations coupled with a three years' course, although he is careful to explain that he does not suggest that "examination for entrance to hospital service is desirable." He alludes also to the educational scheme inaugurated by the Incorporated Association of Hospital Officers, and gives certain examples of subjects on which "the junior officer will welcome tuition." It should be added that inquiries as to the educational scheme of the Incorporated Association of Hospital Officers (which is not restricted to members of the Association) should be addressed to Mr. A. H. Coughtrey, the hon. secretary to the sub-committee, at St. Bartholomew's Hospital, London, E.C. In the meantime, until that scheme is sufficiently ripe for public discussion, Mr. Watts' article puts forward suggestions which invite the opinions of the hospital world.

### DEATH OF MR. ALEXANDER HAYES.

We regret to announce that Mr. Alexander Hayes died on Tuesday night at the early age of fifty-one. His death is understood to be due to some recurrence of the serious internal trouble for which he was operated upon two or three years ago. As joint honorary secretary of the British Hospitals Association, and secretary of the Metropolitan Convalescent Institution, a post which he had held for twenty years, Mr. Hayes was well known to a large circle of hospital officers. Since 1905, the year of its foundation, he had also been secretary to the Convalescent Homes Association, whose objects are to co-ordinate the work of convalescent homes receiving patients from London, and to be a distributary centre of information on the needs of London in respect of convalescent relief. In chapter vii. of "Burdett's Hospitals and Charities" for the year 1914 there was an interesting summary of a paper written by Mr. Hayes on the history and genesis of the convalescent home movement, which his great experience made authoritative, although certain of the deductions which he made are, perhaps, open to criticism. Previous to his appointment as secretary to the Metropolitan Convalescent Institution twenty years ago, Mr. Hayes was assistant in the secretary's office at St. Mary's Hospital. His solid merits and engaging politeness of manner endeared him to many, who will sincerely condole with Mrs. Hayes and her two children in the loss which they have sustained by his death.

### THIS WEEK'S DRUG MARKET.

The general upward tendency of prices is maintained and in the case of some drugs is rather more pronounced. The effect of the suspension of the trade mark "Aspirin" seems to have been to increase the demand for acetylsalicylic acid; now that the word "Aspirin" can be applied to acetylsalicylic acid from whatever source, the drug, especially in tablet form, is being more freely advertised, and possibly consumed in larger quantities; the price has advanced, which is not unnatural in view of the limited stocks. Salicylic acid and salicylate of soda are also dearer again, and until the market is relieved by the expected supplies from France and a considerable increase in the output of British makers, the advancing tendencies will probably continue. It seems by no means unlikely that one of the effects of the promised stoppage of Germany's export trade will be to prevent supplies of synthetic drugs reaching us from the United States; hitherto small consignments of such drugs have reached us from America, but in future that country will very probably have none to spare. Cod-liver oil is again dearer, notwithstanding that the yield of oil from the Norwegian fishings has been good and, in fact, larger than it had been up to a corresponding date last year; the main cause of the advance is the continued large buying of the oil for Germany. Cocaine still tends upwards in price. The position of opium has not greatly changed, and makers of morphine continue very busy.

### TO OUR READERS.

Contributions are specially invited from any of our readers to these columns. They should deal with topical subjects and news. They must be authenticated for the information of the Editor only. The minimum payment if published is 5s. There is no hard-and-fast rule as to space, but notes of about twenty lines in length are preferred.

